# Eight-Section Brocade Exercises Improve the Sleep Quality and Memory Consolidation and Cardiopulmonary Function of Older Adults With Atrial Fibrillation-Associated Stroke

**DOI:** 10.3389/fpsyg.2019.02348

**Published:** 2019-10-22

**Authors:** Wei Lv, Xinxin Wang, Jia Liu, Ping Yu

**Affiliations:** ^1^Department of Cadre Ward, The First Hospital of Jilin University, Changchun, China; ^2^Department of Cardiovascular Medicine, The First Hospital of Jilin University, Changchun, China

**Keywords:** eight-section brocade, sleep, atrial fibrillation-associated stroke, Pittsburgh sleep quality index, memory for word pairs, memory consolidation, cardiopulmonary function

## Abstract

**Purpose:** Poor sleep quality is harmful for everyone and potentially even more harmful for older adults with atrial fibrillation-associated stroke (AFAS). This study aims to explore the effects of eight-section brocade (ESB) on sleep quality, memory, and cardiopulmonary function in the older adults with AFAS.

**Methods:** Older adults with AFAS and sleep disorders were recruited and divided into the ESB (EG, *n* = 85) and control groups (CG, general exercise, *n* = 85). EG patients received 60 min ESB exercises 5 times a week for 12 weeks; CG patients received normal exercise. Pittsburgh sleep quality index (PSQI) scores (poor sleepers ≥8 and normal sleepers < 8), memory for word pairs (poor memory ≤ 7 and normal memory > 7), left ventricular posterior wall (LVPW) thickness, and maximum ventilation (MV, to evaluate cardiopulmonary function) values were measured. The correlation between sleep and memory quality was analyzed using PSQI scores and word pairs via the Pearson correlation coefficients test. Adjusted Cox models were used to explore an interaction between PSQI and ESB exercise.

**Results:** After 12-week exercise intervention, ESB improved sleep quality, latency, duration, disturbance and daytime dysfunction when compared to conventional exercise. In similar cases, the MV values in the EG were also higher than that in the CG (*p* = 0.009). ESB intervention could not affect the cardiac structure and left ventricular ejection fraction. Compared with the CG, the ESB intervention reduced PSQI scores and increased memorized word pairs (*p* < 0.001 for poor and normal sleepers in both unadjusted and adjusted analysis, *p* = 0.012 and 0.003 for poor and normal memory). The test of Pearson correlation coefficients showed that PSQI scores were strongly associated with the number of word pairs in both unadjusted and adjusted analyses (*p* < 0.0001).

**Conclusion:** Eight-section brocade exercise improved sleep quality and memory consolidation and cardiopulmonary function by reducing PSQI scores, increasing word pairs and MV values in the older adults with AFAS.

## Introduction

As a common disease, stroke severely threatens human health and life. The stroke disability rate is extremely high, and numerous patients show different levels of incapacity and lack the ability to take care of themselves, thus causing heavy burdens on themselves, their families, and society (Chhabra et al., 2019). The fundamental method to reduce the incidence, mortality, recurrence rates, and the degree of disability, is to prevent stroke risk. Epidemiological survey data show that the main risk factors of stroke include heart disease (Yuan et al., 2017), hypertension (Morrison and Filosa, 2019), diabetes (Gonzalez-Pascual and Barea, 2019), obesity (Winter et al., 2016), and lack of exercise (Lee et al., 1999). Heart disease can directly increase the risk of stroke. Patients with atrial fibrillation can exhibit higher risks of stroke compared to normal people (Ingall, 2004). Psychological factors have shown that mood (Pappadis et al., 2018), stress (Baune and Aljeesh, 2006), and anxiety (Le Danseur et al., 2019) are also related to stroke occurrence. Improved heart and lung functions positively affect stroke prevention. Stroke prevention mainly comprises drug treatment (Hermann et al., 2019), acupuncture (Sun and Wu, 2019), and exercise (Zheng et al., 2019); the side effects of various drug therapies have also become a causative factor (Alhaboob et al., 2014). Therefore, scholars are eager to find a safe and effective natural therapy for stroke.

The American Heart Association emphasizes the importance of physical exercise in prevention and treatment of cardiovascular and cerebrovascular diseases, whereas regular aerobic exercise can cause a 45.9% risk of death in chronically ill patients (Shen et al., 2016). Therefore, determining a suitable exercise for older adults and prevention of stroke is especially important.

Sleep disorder is a common symptom in older adults with atrial fibrillation-associated stroke (AFAS) (Valenza et al., 2014). Sleep disorders are often accompanied by various psychological problems, such as depression (Paudel et al., 2018; Albuquerque et al., 2019), anxiety (Botokeky et al., 2019), and comorbid insomnia (Tasbakan et al., 2018), which further increase the burden of patients and affect their daily life and learning. Sleep quality is closely related to physical fitness, whereas long-term sleep disorders severely affect patient health. Sleep plays an important role in affecting and regulating mood and maintaining normal emotional memories (Cellini et al., 2019a; Gerhardsson et al., 2019; Jones et al., 2019; Lipinska et al., 2019). On the other hand, patients with sleep disorders are at risk for declarative memory deficits (Cassidy et al., 2017).

Both obstructive sleep apnea (OSA) and atrial fibrillation (AF) can affect ischemic stroke. Nocturnal hypoxia due to OSA is an independent indicator of AFAS in patients with ischemic stroke (Chen et al., 2017). AFAS is the most common arrhythmia that affects the quality of life, causing poor sleep quality, which results in the increase of symptoms (including stroke) in the AFAS patients (Szymanski et al., 2014). On the other hand, poor sleep quality is associated with an increased risk of AFAS (Morovatdar et al., 2019), and AFAS increases stroke risk and adversely affects cardiovascular function (Mead et al., 2005).

At present, drugs (Fereshtehnejad et al., 2018) and cognitive behavioral therapy (Espie et al., 2019) are the main treatment mode for sleep disorders and memory deficits. Although drug treatment of sleep disorders is exact and effective, resulting adverse reactions and drug dependence are notable, and symptoms easily rebound after stopping drug use. Therefore, doctors and scholars are exploring non-medical interventions for sleep disorders and measures to promote physical health. Exercise is an effective way to improve sleep quality and promote health (Purani et al., 2019) and memory function (Loprinzi et al., 2019).

Eight-section brocade (ESB, Chinese name Baduanjin) is a traditional Chinese Qigong and one of the most widely used methods in traditional Chinese health (Spiegel et al., 2009; Liu S.J. et al., 2018; Yu et al., 2018; Atti et al., 2019; Lu et al., 2019; Xie Y. et al., 2019; Ying et al., 2019). ESB brocade consists of eight separate moderate-intensity aerobic exercises (Liu T. et al., 2018), which are intended to be soothing, natural, and easy-to-learn. ESB exercise is mainly involved with the adjustment of mind and breathing, and people can achieve a healthy and harmonious state of mind. The liver, lung, spleen and stomach, heart, kidney, and other organs are properly adjusted, and the head, shoulders, and waist are also adjusted. Exercises are also performed in various body parts, such as the chest and abdomen. Modern research shows that practicing ESB can positively improve heart and lung functions and body shape, balance, flexibility, limb strength, and improves sleep quality in older adults (Zou et al., 2018). ESB strengthens the body, promotes blood circulation, and coordinates internal organ interaction (Zheng et al., 2016; Ying et al., 2019). ESB remarkably improves the respiratory and circulatory systems (Liu S.J. et al., 2018; An et al., 2019; Li et al., 2019; Ying et al., 2019). Furthermore, ESB has been found to improve sleep quality in older adults (Chen et al., 2012) and may exert beneficial effects on older adults with AFAS.

According to the above information, ESB may have protective functions against AFAS in older adults by improving sleep quality, memory ability, and cardiopulmonary function. Poor sleep is harmful for everyone and potentially even more harmful for older adults with AFAS. Therefore, we aimed to explore the effects of ESB on sleep quality, memory and cardiopulmonary function in older adults with AFAS.

## Materials and Methods

### Study Design

A prospective randomized parallel and double-blind study design was used to evaluate the effects of ESB exercise on sleep quality in older adults with AFAS, to examine the relationships of exercise and changes in the Pittsburgh sleep quality index (PSQI) scores.

### Setting and Participants

From 1 March 2016 to 1 March 2017, older adults with AFAS were recruited at The First Hospital of Jilin University (Changchun, China). Atrial fibrillation was measured using intracardiac electrogram recordings, and color tissue Doppler imaging was measured using pulsed ultrasound Doppler and M-mode echocardiography (Lang-Jensen et al., 1983). The effect size of the population size was determined from each of Cohen’s guideline points. The sample size was determined by running a power test for power at 0.9 and α = 0.05. The required population size was *N* = 170 or 85 per group.

The following inclusion criteria were used. Older adult populations with AFAS were evaluated according to the following risk factors: history of hypertension (l40/90 mmHg), smoking, and blood lipids; diabetes; overweightness or obesity; history of cerebral ischemic attack: no regular physical exercise in the past year (2–3 times a week, >30 min each time, for more than 3 months); 55–70 years old. The patients provided written consent forms and volunteered to participate in the experiment. The patients were excluded if they met one of the following criteria: severe organ failure, musculoskeletal disorders, exercise contraindication, and difficulty in communicating; The participants occasionally took hypnotics (such as benzodiazepines, non-benzodiazepine receptor agonists, and melatonin receptor agonists so on) to alleviate their sleep problems within a recent month; Exercise contraindication was identified by ESB tutors if the patients had shortness of breath, severe headache and sudden onset of numbness or weakness after light activity; Several patients failed to follow training requirements and one case showed vomiting and a sudden feeling of weakness during the intervention and thus could not continue to participate. These subjects voluntarily withdrew from the study. The members in ESB group (EG) practiced ESB during the intervention.

Before the experiment, the sleep status test of older adults with AFAS was performed using PSQI (Zhang et al., 2016), and 170 older adults with AFAS with PSQI > 5 served as poor sleep quality and research subjects. These patients were randomly divided into the EG group and the CG group (conventional exercise) using a computer-generated code. All patients showed no family history of mental illness, neuropathy, sleep, endocrine disease, and/or memory disorders.

### Ethics Statement

The study was approved by the Human Institutional Ethics Committee of The First Hospital of Jilin University (Approval No, 20150510C). The articipant’s rights were explained during the interview. These rights include anonymity, confidentiality, privacy, self-determination regarding voluntary participation, ability to withdraw from the study, and interview recording. After verbal and written information were given, informed consent was obtained from all 170 participants prior to the study.

### Data Collection

#### Exercise Intervention

During the experiment, all subjects were banned from using all hypnotic drugs. In the EG, the patients received ESB exercise for 60 min, including 10 min of preparation time, to practice 5 times per week for 12 weeks, in accordance with a previous report (Wang et al., 2008; Liu et al., 2015). In the CG, the patients received normal exercises, that is, walking or brisk walking, without special sports training.

#### Follow-Up Analysis

The follow-up period was 12 weeks, while participants resumed their previous lifestyle. Follow-ups *via* telephone calls were performed every 2 weeks and the main inquiries included changes in lifestyle and condition (Moon et al., 2018).

#### Analysis of Clinical Baseline Characteristics

The following information was investigated: demographic data, gender (male/female), age (years), ethnicity, occupation, education level, marital status, risk factor exposure (such as smoking, history of hypertension, diabetes, and high-level blood lipids), body mass index, and family history; and main indicators of cardiopulmonary function, such as static lung function, including tidal volume, ventilation per mine (VE), maximum ventilation (MV), deep inspiratory volume, vital capacity (VC), forced expiratory volume in 1 s (FEVi), and first-second rate (FEVi/FVC%). Yage sports cardiopulmonary function tester (Yage Technology Co., Ltd., Hamburg, Germany) was used. Cardiac structures and function, including the diameter of the aortic root, main pulmonary artery, left atrial, right atrial, left ventricular diastolic, and right ventricular, thickness of the left ventricular anterior wall, left ventricular posterior wall (LVPW), ventricular septal, and left ventricular ejection fraction (LVEF), were evaluated using the PHILIPS IU22 color Doppler ultrasound imaging system (Avante Health Solutions company, Charlotte, NC, United States).

#### Sleep Quality Test

Before and after the experiment, all subjects were evaluated using PSQI scores. Evaluation indicators included sleep quality, sleep latency, sleep efficiency, and sleep disturbance, use of medicine, and daytime dysfunction. Each factor was scored on a scale of 0 to 3. The cumulative scores of each factor represent the total PSQI scores. PSQI scores ranged from 0 to 21, and a higher score indicated poorer sleep quality. Patients with a global score of >5 were regarded as poor sleepers, and those with a score of 5 or less were regarded as normal sleepers. Sensitivity analyses for PSQI scores between the CG and EG groups were performed after the adjustment for clinical outcomes stratified by PSQI scores. According to the previous report, all patients were assigned as normal sleepers (PSQI < 8) and poor sleepers (PSQI ≥ 8) according to global scores of PSQI (Barakat et al., 2019).

#### Memory Consolidation Training and Test

In the practice period, all participants should learn four pairs of related words displayed on a memory screen in accordance with instructions. The word order is random and should be learned four times. At the end of the study, the participants were asked to follow the instruction and speak the memorized words. The correct rate was 60%; otherwise, participants were asked to continue learning and memorizing. At the end of the practice phase, participants entered the memory period. The participants studied 46 pairs of words, which were randomly presented in accordance with experimental instructions. After a sequence was presented, the participants would recall the words and wrote down the word pairs corresponding to all the words. The correct word pairs were recorded. According to the memorized word pairs, half of the participants were arbitrarily regarded as having normal memory and the other half as having worse memory. Thus, memorized word pairs ≤ 7 were defined as having a worse memory and the memorized word pairs >7 were defined as having normal memory, according to the baseline data.

#### Data Analysis

Statistical analysis was performed using SPSS18.0 statistical analysis software. All data were described by mean values ± standard deviation or median and P25–P75 (median, 25–75%). All statistical tests were performed using two-sided *T*-test, rank sum (for measured data), or χ^2^ tests (for count data). *p* < 0.05 was considered statistically significant. Demographic data and other baseline values were compared between the two groups. Adjusted Cox proportional hazard models were used to assess the association between ESB and clinical outcome, stratified by baseline PSQI including age, gender, job, smoking and drinking habits, and being overweight (Nishinoue et al., 2012).

According to previous reports, sleep quality had a strong impact on memory consolidation (Mignot, 2008). The association of both sleep quality (PSQI scores) and memory consolidation (word pair) was evaluated using the Pearson’s correlation coefficient in adjusted and unadjusted analyses.

## Results

### Baseline Characteristics Between Two Groups

We chose the baseline characteristics [age (Hokett and Duarte, 2019; Scullin et al., 2019), gender (Spencer, 2008), high blood pressure (Borda et al., 2019; Kumar et al., 2019), high blood lipids (Reijmer et al., 2009; Kruisbrink et al., 2017), diabetes (Margolis et al., 2019; Zhu et al., 2019), being overweight (Mora-Gonzalez et al., 2019; Yeo et al., 2019), atrial fibrillation (Takii et al., 2016; Yeung et al., 2019), smoking (Blaes et al., 2019; Cohen et al., 2019), family history of stroke (Baumann et al., 2012; Reeves et al., 2014), and history of transient ischemic attack (Takahashi et al., 2009; Sico et al., 2017)] according to previous reports indicating that these parameters would affect sleep and memory. The statistical difference for baseline characteristics was insignificant between the two groups (Table 1, *p* > 0.05). The results suggested that baseline characteristics would not affect subsequent analysis. One-hundred-and-seventy participants were equally assigned into the EG and CG groups (*n* = 85 for each group). One participant dropped out during follow-up in the EG and CG groups after intervention, respectively. Six and 16 participants dropped out after the 12-week follow-up in the EG and CG groups (retention rate: 92.9 and 81.2%), respectively.

**TABLE 1:** Clinical baseline characteristics between two groups.

**Parameters**	**EG (*n* = 85)**	**CG (*n* = 85)**
Age (y)	60.53 ± 5.29	59.75 ± 4.34
Gender (Male/Female)	31/54	30/55
High blood pressure (N/Y)	39/46	42/43
High blood lipids (N/Y)	18/67	23/62
Diabetes (N/Y)	68/17	68/17
Overweight (N/Y)	29/56	35/50
Atrial fibrillation (N/Y)	84/1	81/4
Smoking (N/Y)	70/15	77/8
Drinking (N/Y)	55/40	57/38
Job (Full-time/Part-time)	68/17	65/20
Family history of stroke (N/Y)	53/32	55/30
History of transient ischemic attack (N/Y)	67/18	67/18

*EG, eight-section brocades exercise group. GG, general exercise group. The age was presented as mean values ± *SD* (Standard deviation). The statistical difference was insignificant for all parameters between two groups.*

### ESB Had No Effect on Cardiac Structure and Left Ventricular Ejection Fraction (%)

Before intervention and after analyzing the results of cardiac ultrasound by *T*-test, results showed no significant difference for cardiac structure and left ventricular ejection fraction between the EG and CG (*p* > 0.05). After intervention, echocardiography results were analyzed by *T*-test. No significant difference was observed between the EG and CG (*p* > 0.05). At the end of follow-up, heart color ultrasound was compared by *T*-test, and the difference between the two groups was insignificant (*p* > 0.05, Table 2). LVPW thickness in the EG decreased after ESB intervention and follow-up, but the difference was also insignificant (*p* = 0.885, Table 2).

**TABLE 2:** Comparison of cardiac structure and left ventricular ejection fraction (%) between two groups.

**Parameters**	**Time**	**ESB**		**CG**		***t/z***	***P***
		***n***		***n***			
Septal thickness (cm)	Baseline	85	1.01 ± 0.12	85	1.02 ± 0.11	0.919	0.360
	Intervention	84	0.98 ± 0.11	84	0.98 ± 0.11	–0.007	0.994
	Follow-up	78	0.96 ± 0.12	68	0.97 ± 0.10	0.312	0.755
LVPW thickness (cm)	Baseline	85	0.9 ± 0.09	85	0.9 ± 0.09	–0.433	0.666
	Intervention	84	0.93 ± 0.08	84	0.94 ± 0.09	1.298	0.196
	Follow-up	78	0.92 ± 0.11	68	0.93 ± 0.09	0.145	0.885
LVAW thickness (cm)	Baseline	85	1.0 ± 0.12	85	1.0 ± 0.13	0.618	0.537
	Intervention	84	0.98 ± 0.09	84	0.98 ± 0.10	–0.064	0.949
	Follow-up	78	0.97 ± 0.09	68	0.96 ± 0.08	–0.547	0.585
Left ventricular ejection fraction (%)	Baseline	85	62.29 ± 4.35	85	61.92 ± 3.77	–0.603	0.548
	Intervention	84	63.49 ± 4.00	84	63.19 ± 4.59	–0.507	0.612
	Follow-up	85	2.80 ± 0.30	85	2.83 ± 0.31	0.569	0.570
Aorta internal passage (cm)	Baseline	84	2.92 ± 0.34	84	2.91 ± 0.39	–0.219	0.827
	Intervention	78	2.86 ± 0.31	68	2.85 ± 0.36	–0.063	0.950
	Follow-up	85	3.22 ± 0.40	85	3.18 ± 0.40	–0.697	0.487
Left atrial diameter (cm)	Baseline	84	3.30 ± 0.38	84	3.22 ± 0.33	–1.419	0.158
	Intervention	78	3.14 ± 0.43	68	3.10 ± 0.39	–0.656	0.513
	Follow-up	85	4.45 ± 0.42	85	4.38 ± 0.44	–1.028	0.306
Left ventricular diastolic diameter (cm)	Baseline	84	4.41 ± 0.33	84	4.41 ± 0.37	0.095	0.925
	Intervention	78	4.43 ± 0.34	68	4.39 ± 0.41	–1.041	0.302
	Follow-up	85	3.16 ± 0.28	85	3.11 ± 0.32	–1.032	0.304
Right atrial diameter (cm)	Baseline	84	3.12 ± 0.34	84	3.07 ± 0.35	–1.046	0.297
	Intervention	78	3.09 ± 0.34	68	3.08 ± 0.35	–0.267	0.790
	Follow-up	85	2.97 ± 0.34	85	2.99 ± 0.30	0.613	0.504
Right indoor diameter (cm)	Baseline	84	2.99 ± 0.36	84	2.92 ± 0.34	–1.708	0.089
	Intervention	78	2.83 ± 0.34	68	2.81 ± 0.33	–0.338	0.736
	Follow-up	85	2.04 ± 0.20	85	2.05 ± 0.24	0.238	0.812
Main pulmonary artery diameter (cm)	Baseline	84	2.09 ± 0.20	84	2.07 ± 0.17	–0.392	0.696
	Intervention	78	2.11 ± 0.21	68	2.08 ± 0.19	–0.743	0.459
	Follow-up	85	2.80 ± 0.30	85	2.83 ± 0.31	0.569	0.570

*EG, eight-section brocades exercise group. CG, general exercise group. LVPW, left ventricular posterior wall. LVAW, left ventricular anterior wall. The data were presented as mean values ± *SD* (Standard deviation). The statistical difference was significant if *p* < 0.05 vs. the CG group.*

### ESB Increased MV Values

Maximum ventilation values are often applied to measure cardiopulmonary function (Jang et al., 2017; Kim et al., 2018). Results of the study showed no significant difference for MV values between the EG and CG before intervention (*p* = 0.464). After intervention, the statistical difference for MV values was insignificant after the T- or non-parametric test (*p* = 0.367). At the end of the follow-up period, the EG showed higher MV values than the CG (*p* = 0.009, Table 3). In terms of MV, the EG showed an upward trend compared with the CG (Table 3). The statistical difference for other parameters of lung function was insignificant in all periods (*p* > 0.05, Table 3).

**TABLE 3:** The effects of eight-section brocades exercise on lung function.

**Paramters**	**Time**	**EG**	**CG**	***p*-values**
Tidal volume (L)	Baseline	0.59 (0.45–0.74)	0.57 (0.43–0.76)	0.647
	Intervention	0.69 (0.53–0.83)	0.68 (0.50–0.88)	0.862
	Follow-up	0.62 (0.45–0.82)	0.60 (0.46–0.79)	0.977
Ventilation per minute (L/min)	Baseline	9.87 (8.10–13.22)	10.05 (7.96–13.50)	0.836
	Intervention	10.79 (7.94–16.00)	10.59 (8.50–15.29)	0.992
	Follow-up	11.12 (8.77–15.33)	10.96 (8.08–13.35)	0.211
Vital capacity (L)	Baseline	3.02 ± 0.63	2.95 ± 0.70	0.454
	Intervention	3.09 ± 0.65	3.02 ± 0.66	0.490
	Follow-up	3.09 ± 0.65	3.00 ± 0.67	0.817
Maximum ventilation (L/min)	Baseline	88.01 ± 26.83	85.03 ± 25.02	0.464
	Intervention	88.79 ± 26.14	85.44 ± 2136	0.367
	Follow-up	100.37 ± 21.28	92.30 ± 14.80	0.009
Forced expiratory volume in the first second (L)	Baseline	2.46 ± 0,54	2.37 ± 0.52	0.292
	Intervention	2.51 ± 0.52	2.39 ± 0.46	0.135
	Follow-up	2.49 ± 0.51	2.47 ± 0.51	0.821
1st second rate (%)	Baseline	87.79 ± 6.36	88.14 ± 6.46	0.721
	Intervention	84.31 ± 6.27	82.62 ± 6.70	0.096
	Follow-up	83.67 ± 6.73	83.34 ± 6.74	0.772
Deep inspiratory volume (L)	Baseline	2.40 ± 0.43	2.38 ± 0.55	0.853
	Intervention	2.50 ± 0.48	2.45 ± 0.51	0.541
	Follow-up	2.52 ± 0.54	2.51 ± 0.57	0.952

*EG, eight-section brocades exercise group. CG, general exercise group. The data were presented as median (min–max) or mean values ± *SD* (Standard deviation). *n* = 84, 84, and 79 in the EG group and 81, 82, and 69 in the CG group before intervention, after intervention and at 12-week follow-up, respectively. *n* = 76, 76 and 74 in the EG group and 76, 64, and 65 in the CG group for total scores before intervention, after intervention and at 12-week follow-up, respectively. The statistical difference was significant if *p* < 0.05 vs. the CG group.*

### ESB Improved Sleep Quality

Pittsburgh sleep quality index was used to assess participants’ sleep quality. Prior to intervention, PSQI scores were non-parametric and exhibited no significant differences between the two groups (*p* > 0.05, Table 4). After intervention, ESB intervention improved sleep quality, including subjective sleep quality, latency, persistence, and disturbance, daytime dysfunction, and the total sleep score, compared with the CG (*p* < 0.001 in both unadjusted and adjusted analyses, respectively, Table 4). No statistically significant difference was found between the groups in terms of sleep efficiency and medication (Table 4). At the end of the follow-up period, the ESB intervention yielded lower PSQI scores for subjective sleep quality, latency, persistence, and disturbance, daytime dysfunction, and total sleep scores when compared to the conventional exercise (*p* < 0.001 or = 0.001 in both unadjusted and adjusted analyses, respectively, Table 4). No significant difference was observed between the two groups in terms of habitual sleep efficiency and the use of sleep drugs (Table 4). These results suggest that the EG performed better than the CG in improving sleep quality, latency, duration, and disturbance, and daytime dysfunction and showed a sustainable effect.

**TABLE 4:** The effects of ESB on PSQI (Median, 25–75%).

		**EG**	**CG**	**Unadj. *P***	**EG**	**CG**	**Adj. *p***
Sleep quality	Baseline	1 (1–2)	1 (1–2)	0.975	1 (1–2)	1 (1–2)	0.918
	Intervention	1 (1–1)	1 (1–2)	<0.001	1 (1–1)	1 (1–2)	<0.001
	Follow-up	1 (1–1)	1 (1–2)	<0.001	1 (1–1)	1 (1–2)	<0.001
Sleep latency	Baseline	2 (1–2)	2 (1–3)	0.567	2 (1–2)	2 (1–3)	0.362
	Intervention	1 (1–2)	2 (1–2)	<0.001	1 (1–2)	2 (1–2)	<0.001
	Follow-up	1 (1–2)	2 (1–2)	0.001	1 (1–2)	2 (1–2)	<0.001
Sleep duration	Baseline	1 (1–2)	1 (1–1)	0.326	1 (1–2)	1 (1–1)	0.241
	Intervention	1 (1–2)	2 (1–2)	<0.001	1 (1–2)	2 (1–2)	<0.001
	Follow-up	1 (1–2)	2 (1–2)	0.002	1 (1–2)	1 (1–2)	0.001
Sleep efficiency Sleep efficiency	Baseline	1 (0–1)	1 (0–1)	0.768	1 (0–1)	1 (0–1)	0.486
	Intervention	1 (0–1)	1 (0–1)	0.351	1 (0–1)	1 (0–1)	0.139
	Follow-up	1 (0–1)	1 (0–1)	0.991	1 (0–1)	1 (0–1)	0.812
Sleep disturbance Sleep disturbance	Baseline	1 (1–2)	1 (1–2)	0.605	1 (1–2)	1 (1–2)	0.579
	Intervention	1 (0–4)	2 (1–2)	<0.001	1 (0–4)	2 (1–2)	<0.001
	Follow-up	1 (1–1)	2 (1–2)	<0.001	1 (1–1)	2 (1–2)	<0.001
Use of Medication Use of Medication	Baseline	0 (0–0)	0 (0–0)	0.632	0 (0–0)	0 (0–0)	0.531
	Intervention	0 (0–0)	0 (0–0)	0.501	0 (0–0)	0 (0–0)	0.417
	Follow-up	0 (0–0)	0 (0–0)	0.578	0 (0–0)	0 (0–0)	0.328
Daytime dysfunction Daytime dysfunction	Baseline	1 (0–1)	0 (0–1)	0.813	1 (0–1)	0 (0–1)	0.533
	Intervention	0 (0–1)	1 (0–1)	0.001	0 (0–1)	1 (0–1)	<0.001
	Follow-up	0 (0–1)	0 (0–1)	0.017	0 (0–1)	0 (0–1)	<0.001
Total scores	Baseline	7 (5–9)	7 (5–10)	0.703	7 (4–9)	7 (5–9)	0.584
	Intervention	5 (4–7)	8 (6–10)	<0.001	5 (4–7)	8 (6–9)	<0.001
	Follow-up	5 (4–7)	8 (6–10)	<0.001	5 (4–7)	8 (6–10)	<0.001

*Adjusted Cox proportional hazards models to assess the association between ESB and PSQI scores by baseline PSQI, including age, gender, job, smoking, drinking and overweight. EG, eight-section brocades exercise group. CG, general exercise group. PSQI, Pittsburgh sleep quality index. A higher PSQI score indicates poorer sleep states. *n* = 85, 84, and 79 in the EG group and 85, 84, and 69 in the CG group before intervention, after intervention and at 12-week follow-up, respectively. The statistical difference was significant if *p* < 0.05 vs. the CG group.*

**FIGURE 1:**
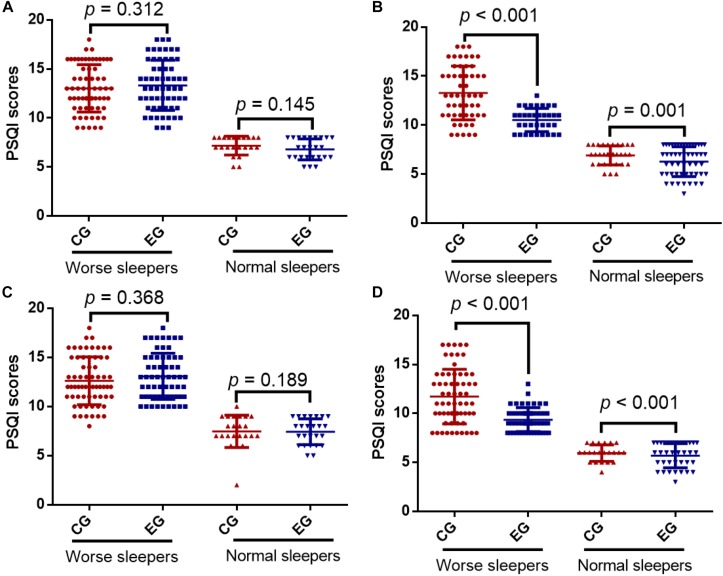
Pittsburgh Sleep Quality Index (PSQI) scores between the control group (CG) and the eight-section brocades (ESB) exercise group (EG). **(A)** Before intervention in unadjusted analysis. **(B)** After 12-week intervention in unadjusted analysis. **(C)** Before intervention in adjusted analysis. **(D)** After 12-week intervention in adjusted analysis. Adjusted Cox proportional hazards models to assess the association between ESB and clinical outcomes by baseline PSQI, including age, gender, job, smoking and drinking habits and overweight (53). PSQI < 8 was regarded normal sleepers and PSQI ≥ 8 was designed as worse sleepers. *n* = 85 for each group and the statistical difference was significant if *p* < 0.05.

Sensitivity analysis results indicated that PSQI scores were reduced in the both groups after adjustment but the reduction in the EG group was more than in the CG group. After adjustment, the statistical difference remained significant (Table 4), suggesting that the results were robust. Before intervention, the number of worse (63 cases and 60 cases in the CG and EG groups, respectively) and normal sleepers (22 case and 25 cases in the CG and EG groups, respectively) was almost the same, and the values of worse and normal sleepers were similar between the two groups before adjustment (Figure 1A, *p* = 0.312 and 0.145). After the follow-up, the number of worse sleepers (34 cases) was reduced and the number of normal sleepers (51 cases) was increased in the EG group higher than in the CG groups (53 and 32 cases). The PSQI values of worse and normal sleepers in the CG group were higher than in the EG group before adjustment (Figure 1B, *p* < 0.001 or = 0.001). In the cases that were similar, before the exercise intervention, the values of worse and normal sleepers were similar between the two groups after adjustment (Figure 1C, *p* = 0.368 and 0.189). After the 12-week follow-up, the PSQI values of worse and normal sleepers in the CG group were higher than in the EG group before adjustment (Figure 1D, *p* < 0.001 or = 0.002). ESB exercise improved the sleep quality when compared to conventional exercise.

#### ESB Improved Memory for Word Pairs

**FIGURE 2:**
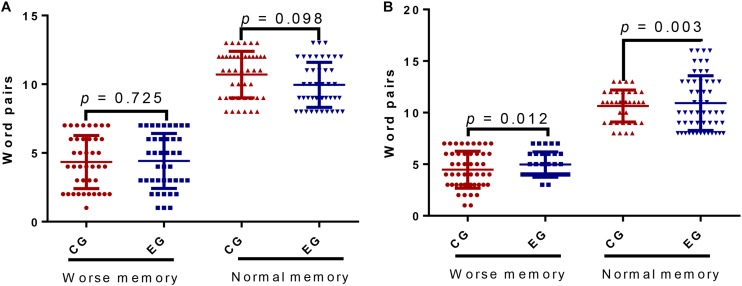
The memory for word pairs between the control group (CG) and the ESB exercise group (EG). **(A)** Before intervention. **(B)** After 12-week intervention. Pair words ≤ 7 was regarded worse memory and pair words >7 was designed as normal memory. *n* = 85 for each group and the statistical difference was significant if *p* < 0.05.

Before intervention, the number of patients with worse and normal memory (41/41 and 44/44 cases) was same, and word-pair values of worse and normal memory were similar between the two groups before adjustment (Figure 2A, *p* = 0.725 and 0.098). After follow-up, the number of the patients with worse memory (32 cases) was reduced and normal memory (53 cases) was increased in the EG group in the CG groups (49 and 36 cases). The word-pair values of worse and normal memory in the EG group were lower than in the CG group (Figure 2B, *p* = 0.012 or = 0.003).

#### Sleep Quality Was Strongly Associated With Memory Quality

**FIGURE 3:**
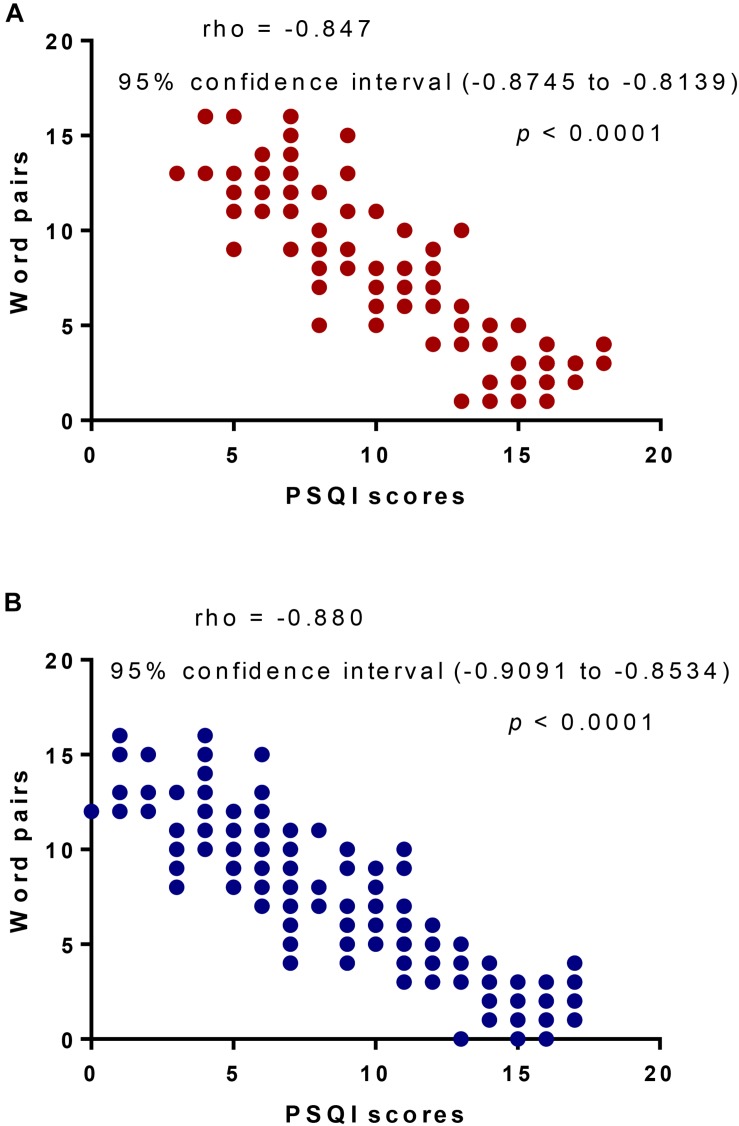
The analysis of Pearson correlation coefficients between PSQI scores and word pairs. **(A)** Unadjusted analysis. **(B)** Adjusted analysis. Adjusted Cox proportional hazards models to assess the association between word pairs and PSQI scores by baseline PSQI, including age, gender, high blood pressure, high blood lipids, diabetes, overweight, AFAS, smoking and family history of stroke. There will be a strong negative relation if rho value falls within -0.5 and -1.

Pittsburgh sleep quality index scores had a strong negative relationship with word pairs before adjustment (Figure 3A, *p* < 0.0001) and similar significant results were also observed after adjustment (Figure 3B, *p* < 0.0001). The results suggested that sleep quality was strongly associated with memory quality. Thus, ESB may improve memory consolidation by reducing PSQI scores.

## Discussion

Eight-section brocade can maintain the stability of LVEF and enhance the contractility of myocardium, thus relieving pressure on the heart and reducing thickness of the LVPW (Mao et al., 2016). The effect of ESB on cardiac function is closely related to the exercise approach (Yu et al., 2018). ESB focuses on movements with the combination of breathing and mind (Li et al., 2015). During slow inhalation, the chest is enlarged, heart and lung compression is relatively reduced, and blood circulation is strengthened (Chen et al., 2016). ESB exhibits certain effects on the cardiac structure and functions (Zheng et al., 2019), however, no significant difference was observed in these improvements. Considering insufficient intervention periods and the generally normal cardiac structure of most of the participants, evident beneficial effects could not be achieved.

Eight-section brocade focuses on adjusting breathing during practice and can relax the body and massage internal organs and can improve lung function and delay the development of lung function damage (Liu S.J. et al., 2018). In this study, no significant difference was observed between the EG and CG before and after intervention. At the end of the follow-up period, a significant difference for MV values was found between the two groups (Table 3). ESB improved lung capacity by increasing MV values when compared to conventional exercise. The results may be associated with breathing exercise of ESB because slow breathing expands the lungs to the maximum capacity.

Sleep is one of the most important physiological activities of humans. Sleep quality exerts an important effect on human health and life. In severe cases, poor sleep can affect immunity (Oikonomou and Prober, 2019), physiological metabolic activities (Spiegel et al., 2009) and memory (Xie W. et al., 2019), and induce hypertension (Chiang et al., 2018) and heart disease (Norra et al., 2012). Sleep quality is closely related to cognitive function (Leng et al., 2017). Sleep quality in older adults is generally poor, and this condition not only affects physical health but also mental condition. ESB exercise can improve the sleep quality of middle-aged and older adults and is an effective method for treating insomnia (Jiang et al., 2017).

This study used PSQI to assess the sleep quality of participants. PSQI consists of subjects, such as subjective sleep quality, sleep latency, persistence, and disturbance, and daytime dysfunction. The cumulative score represents the total PSQI score, and a higher score implies a poorer sleep quality of the subject. After exercise intervention, significant differences were found in PSQI scores between the two groups in terms of subjective sleep quality, sleep latency, persistence, and disturbance, daytime dysfunction, and total sleep score but not in habitual sleep efficiency and use. No statistically significant difference was observed in use of medication between the two groups before and after exercise intervention. After the follow-up period, the same results were also observed. In terms of subjective sleep quality, sleep latency, persistence, and disturbance, daytime dysfunction, and total sleep score, PSQI scores exhibited statistical significance. No significant difference was observed between the two groups in terms of habitual sleep efficiency and use of sleep medication. The improvement effect of ESB on sleep quality is mainly reflected in the enhancement of difficulty with falling asleep and prolonging sleep persistence, and the effect can be sustained until the follow-up period. Notably, though participants were randomly assigned to groups, a sensitivity analysis that controlled for baseline PSQI was performed. After the adjustment, the PSQI scores were reduced in the EG group more than in the CG group when compared to the adjustment. Meanwhile, the *p*-values in the adjustment analysis were lower than those before adjustment. The results suggest that ESB provides an effective method in improving sleep quality of the older adults with AFAS.

The present findings indicate a higher number of memorized word pairs in the EG than in the CG. Improvement of sleep quality showed good effects on consolidation of declarative memory, consistent with a previous report indicating the negative effect of sleep loss on declarative memory consolidation (Cousins and Fernandez, 2019). Good sleep can also improve the correct rate of word recognition (Wang et al., 2017). Sleep plays an irreplaceable role in the process of consolidating declarative memory. Different ideas are available for the mechanism by which sleep reinforces declarative memory, suggesting that the problem still requires further exploration (Cellini et al., 2019b; Cousins and Fernandez, 2019; Lipinska and Thomas, 2019).

The strengths of the present study were that ESB intervention (1) reduced PSQI scores by improving sleep quality, latency, persistence, and disturbance and daytime dysfunction; (2) improved cardiopulmonary function by increasing MV values and (3) improved memory consolidation by increasing memorized word pairs when compared to conventional exercise. There were some limitations in the present study. A 12-week exercise period was a short intervention period, and the effects of long-term ESB should be explored in the future. Approximately ∼170 participants is a small sample size to explore the function of ESB in. Therefore, larger and longer trials are needed to confirm these results.

## Conclusion

Eight-section brocade exercise improved sleep quality, memory consolidation, and MV of older adults with AFAS when compared to conventional exercise and showed a possible beneficial function on their health. Further work should be performed to confirm the exact mechanism of the effects of ESB on older adults with AFAS, and ESB must be tested on a large population sample for a longer period in the future.

## Data Availability Statement

The datasets analyzed in this manuscript are not publicly available. Requests to access the datasets should be directed to JL, liujiajlu@163.com.

## Ethics statement

The studies involving human participants were reviewed and approved by the Human Institutional Ethics Committee of The First Hospital of Jilin University (Approval No. 20150510C). The patients/participants provided their written informed consent to participate in this study.

## Author Contributions

WL developed the concept. XW designed the study. JL performed the data acquisition, supervision, data analysis, and interpretation. PY prepared the manuscript. JL and PY critically revisied the manuscript. Prior to submission, all authors read and approved the final manuscript.

## Conflict of Interest

The authors declare that the research was conducted in the absence of any commercial or financial relationships that could be construed as a potential conflict of interest.
